# Is preoperative core biopsy accurate in determining the hormone receptor status in women with invasive breast cancer?

**DOI:** 10.1186/1477-7800-2-15

**Published:** 2005-08-22

**Authors:** W Al Sarakbi, M Salhab, V Thomas, K Mokbel

**Affiliations:** 1 St George's and The Princess Grace Hospitals, London, UK

**Keywords:** core needle biopsy, oestrogen receptors, progesterone receptors

## Abstract

**Background:**

The objective of this study was to determine the concordance rate between core needle biopsy (CNB) and surgical excision of invasive breast cancer regarding the oestrogen receptor (ER) and Progesterone receptor (PgR) status as determined by Immunohistochemistry (IHC).

**Methods:**

Hormone receptor status was established using IHC (using quickscore system 0–8) on preoperative CNB and subsequent surgical excision in 93 patients with invasive breast cancer. Results were compared taking into account tumour's size, grade, and patient's age.

**Results:**

The ER concordance rate between CNB and surgical excisions was 95%. The PgR concordance rate was 89%. This shows that CNB has a sensitivity of 97% for ER and 95% for PgR.

There is a positive correlation of ER and PgR between CNB and surgical excision (p < 0.000001). There was no significant difference in the number of core biopsies between concordant and discordant cases.

**Conclusion:**

Preoperative core biopsy is highly sensitive for the IHC detection of ER and PgR in invasive breast cancer. The concordance rate is higher for ER than PgR, which could be due to the fact that ER is more homogeneously distributed.

## Introduction

The core needle biopsy (CNB) is technique increasingly used for the preoperative assessment of breast lesions [1]. Image guidance increases accuracy and reduces the number of false negative cases [2]. The presence of malignancy and tumour's type and grade are routinely reported.

There is an increasing need to provide prognostic data on CNB in order to improve treatment outcome [3,4].

Hormone receptor status, and especially ER, provides valuable prognostic information and predicts the response to adjuvant and neo-adjuvant systemic treatment [5].

Traditionally, the hormone receptor status was determined by enzyme immunoassay of ER and PgR proteins. However, this has been replaced gradually by immunohistochemistry (IHC), which has shown an equal reliability [6].

Previous studies that examined the reliability of preoperative CNB using enzyme immunoassay showed conflicting results [7-9].

This retrospective study examines the correlation between CNB and surgical excision in regards to the ER ad PgR status of invasive breast cancer using IHC.

## Patients and Methods

In this retrospective study we looked at consecutive 95 cases of invasive breast carcinoma in 93 patients. All patients underwent CNB at their clinic appointment and proceeded for breast surgery subsequently 2–3 weeks later. Preoperative CNBs and surgical excision specimens were analysed for ER and PgR status using IHC (DAKO mab) after antigen retrieval at high temperature. All specimens were analysed using semi quantitative IHC "quick score" system (0 – 8) by the same breast pathologist. With this method, the intensity of the immunohistochemical reaction as viewed under the light microscope was recorded 0–4 (0 indicated no staining of any nuclei even at high magnification). The proportion of cells staining positively at any intensity was scored as 0 (no cell staining), 1 (1–25% cells stained), 2 (26–50% cells stained), 3 (51–75% cells stained) or 4 (when >75% cells stained). The proportion and intensity scores were added together to obtain a total score ranging from 0 to 8.

We also examined other parameters including: tumour size, grade, and patient's age.

ER/PgR status was considered positive if quick score was 2 – 8. Results were re-assessed when quick score was raised to 4 – 8 to label ER/PgR status as positive. The number of biopsies taken on each occasion was also recorded.

## Results

The mean and median age of this study group was 59.2 and 59 respectively (range 32 – 92 years). The mean tumour size was 20 mm (3.2 – 110 mm), and the median grade was 2 (1 – 3). The median number of CNBs taken at preoperative assessment was 2.6 (1 – 9). The mean IHC score for ER was 6.7 (range 0 – 8).

All patients had either mastectomy or lumpectomy as their definitive surgical treatment.

**Figure 1:**
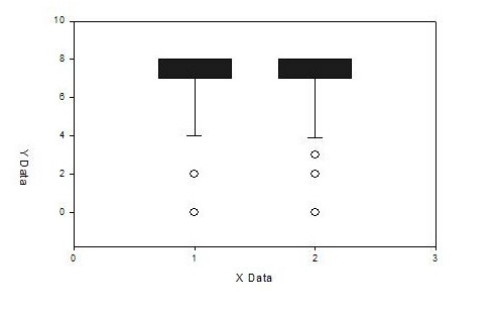
Graphic Results, 1: ER in CNB, 2: ER in surgical excision.

Firstly, ER/PgR status was considered positive if quick score was 2 – 8. The concordance for tumour grade was 65%. The concordance rate for ER was 95% between CNB and surgical excision. There were 2 false negative and 3 false positive cases. PgR concordance rate was 89% with 4 false negative and 6 false positive cases. According to the above results the sensitivity of CNB for ER and PgR was 97% and 95% respectively. Furthermore, to re-examine our findings, we analysed the results considering ER/PgR to be positive if quick score was 4 – 8. We found a concordance rate for ER to be 93% (3 false negative and 5 false positive cases). The concordance rate for PgR was 92% (3 false negative and 6 false positive cases) (figure 1). This gives CNB sensitivity of 98% for positive ER status and 96% for positive PgR status (table 1).

**Table 1:** Sensitivity and specificity of CNB for ER and PgR Status.

**Parameter**	**ER status N = 95**	**PgR status N = 93**
Positive CNB	89	85
Negative CNB	6	8
Positive SE	86	82
Negative SE	9	11
Sensitivity of CNB	97.6%	96.3%
Specificity of CNB	96.6%	96.4%
Concordance rate	92.6%	92.4%

CNB: Core Needle Biopsy

SE: Surgical Excision

Positive: Quickscore 4 – 8

Negative: Quickscore 0 – 3

ER in CNB positively correlated with ER in surgical excision (r: 0.61, p < 0.000001). PgR in CNB also positively correlated with PgR in surgical excision (r: 0.66, p < 0.000001)

There was no significant difference in the number of CNBs between concordant and discordant cases.

## Discussion

Core needle biopsies offer a reliable and accurate assessment of hormone receptor status.

Previous studies on a smaller case sample have suggested similar findings regarding ER [10-12]. However, results on PgR were less consistent.

The concordance rate in our study for ER was higher than for PgR. Homogenous distribution of ER through out the tumour is a possible explanation. Heterogeneity of the ER in tumour cell populations may have important implications for analytic cell selection and for prognosis in ER-positive carcinomas. Previous studies have shown homogenous geographic distribution of ER in the tumour cell population [13,14].

These results indicate that the hormone receptor status as determined by CNB can be reliably used to guide neo-adjuvant and adjuvant systemic therapy in patients with invasive breast cancer.

In summary, preoperative CNB is highly sensitive for the IHC detection of ER and PgR in invasive breast cancer.
